# Potential range shift of a long-distance migratory rice pest, *Nilaparvata lugens*, under climate change

**DOI:** 10.1038/s41598-024-62266-x

**Published:** 2024-05-21

**Authors:** Jinsol Hong, Minyoung Lee, Yongeun Kim, Yun-Sik Lee, June Wee, Jung-Joon Park, Woo-Kyun Lee, Youngil Song, Kijong Cho

**Affiliations:** 1 Ojeong Resilience Institute, Korea University, Seoul, 02841 Republic of Korea; 2 Department of Biological Sciences, Ulsan National Institute of Science and Technology, Ulsan, 44919 Republic of Korea; 3 Department of Biology Education, Pusan National University, Busan, 46241 Republic of Korea; 4 Department of Plant Medicine, Institute of Agriculture and Science, Gyeongsang National University, Jinju, 52828 Republic of Korea; 5 Department of Environmental Science and Ecological Engineering, Korea University, Seoul, 02841 Republic of Korea; 6 Korea Adaptation Center for Climate Change, Korea Environment Institute, Sejong, 30147 Republic of Korea

**Keywords:** Migratory pest, Brown planthopper, Overwintering, Heat stress, Species distribution model, CLIMEX, Climate-change impacts, Projection and prediction, Agroecology, Biogeography, Climate-change ecology

## Abstract

The biogeographical range shift of insect pests is primarily governed by temperature. However, the range shift of seasonal long-distance migratory insects may be very different from that of sedentary insects. *Nilaparvata lugens* (BPH), a serious rice pest, can only overwinter in tropical-to-subtropical regions, and some populations migrate seasonally to temperate zones with the aid of low-level jet stream air currents. This study utilized the CLIMEX model to project the overwintering area under the climate change scenarios of RCP2.6 and RCP8.5, both in 2030s and 2080s. The overwintering boundary is predicted to expand poleward and new overwintering areas are predicted in the mid-latitude regions of central-to-eastern China and mid-to-southern Australia. With climate change, the habitable areas remained similar, but suitability decreased substantially, especially in the near-equatorial regions, owing to increasing heat stress. The range shift is similar between RCP2.6-2030s, RCP2.6-2080s, and RCP8.5-2030s, but extreme changes are projected under RCP8.5-2080s with marginal areas increasing from 27.2 to 38.8% and very favorable areas dropping from 27.5 to 3.6% compared to the current climate. These findings indicate that climate change will drive range shifts in BPH and alter regional risks differently. Therefore, international monitoring programs are needed to effectively manage these emerging challenges.

## Introduction

Climate change has serious impacts on the ecology of insect pests. The warming climate can potentially increase pest population size, geographic distribution and, in particular, enhance overwintering survivability due to increasing winter temperatures^1,2^. Thus, ongoing climate change is expected to alter the geographic distribution of many insect pests, resulting in a range shift (a distribution change in extent and direction) that threatens food security in novel areas^2,3^. With climate warming, tropical insect pests are more likely to survive in temperate zones; however, frequent and extreme summer temperature events can negatively affect their biology. Temperatures that exceed the upper tolerance threshold of an insect species can result in a decrease in growth and reproduction and an increase mortality^4^. However, many recent studies have focused on the expansion of the overwintering boundaries of their target species, and a few have attempted to determine the impact of heat stress caused by temperatures near or above the upper thermal limits^5–7^.

Rice is one of the world's major staple food crops, and is essential for global food security^8^. The brown planthopper (BPH) *Nilaparvata lugens* (Stål) (Hemiptera: Delphacidae), is considered one of the most serious rice pests in the rice-growing regions of Asia^9^. This species is a monophagous vascular feeder that damages plants directly by sucking sap on the phloem from the rice leaf sheath and indirectly by transmitting the rice ragged stunt virus, rice black-streaked dwarf virus, and rice grassy stunt virus^10,11^. The most serious result of BPH infestation is hopper burn in rice fields, which has led to complete yield loss in many Asian countries^12^. BPHs can only overwinter in tropical and subtropical regions such as southern China, the Indochina peninsula, and Southeast Asia which they migrate to every year with the aid of low-level jet stream air currents during the monsoon season^13–15^. Populations from the Indochina peninsula (e.g., the Red River Delta and the Mekong River Delta) are considered the primary source of migratory populations to the temperate regions of East Asia (e.g., China, Korea, and Japan)^13^. This long-distance migration ability is an adaptive behavior seen more in temperate continental areas than in tropical regions^16^. As long-distance migration to temperate zones is solely determined by atmospheric movements during the monsoon season, it is difficult to predict the direction and distance of the migrating BPH population in the future. Thus, understanding the change to the overwintering areas of BPHs, which is the reason for long-distance migration, is a priority when establishing future management strategies.

The overwintering boundary of BPHs is expected to expand owing to the increase in winter temperature under climate change. However, the climatic suitability for BPHs in some areas of the current overwintering area may be reduced by increased heat stress caused by extreme heat events during summer^17^. Thus, climate change can have both positive and negative impacts on BPH populations, but little is known about whether the overall distribution of the BPH will expand or contract as a result of future climate change and to what extent climate change will influence BPH establishment across different geographical regions. However, understanding these aspects of the biogeographical distribution of the BPH remains challenging.

To project the potential range and direction of distribution changes for insect pest species, species distribution models (SDMs) have been proposed as a useful tool and are actively utilized to map the potential geographic distribution of agricultural pest species^7,18,19^. However, the ecological theory and assumptions underlying SDMs are typically inappropriate for long-distance migratory species^20,21^. One of the fundamental SDM assumptions is that the range of the target species is in equilibrium with the current environmental conditions in its native range^22^. However, in a real environment, the native range is restricted by biotic interactions (e.g., competition, predation, and human disturbance) and dispersal limitations^23^. In this respect, the application of typical SDMs to long-distance migratory species clearly violates the underlying assumptions of SDM as the species can avoid biophysical limitations, including geographical barriers that must be overcome to complete their life cycles by means of migration. BPHs can passively migrate from focal overwintering regions to temperate regions. That is, they can avoid exposure to cold winter temperatures, which makes overwintering in temperate zones impossible, and can seasonally invade, repeating the expansion and retraction of their geographic range^13^. As a consequence of ignoring the equilibrium assumptions in SDMs, we believe that the conceptualization of potential versus actual distribution is often confused when using SDMs for long-distance migratory species.

SDMs are categorized into correlative and mechanistic approaches^24^. Correlative SDMs depend completely on the correlation between occurrence records and environmental variables. MaxEnt is the best-known model in this approach. One disadvantage of this model is that the exact implementation heavily relies on the quantity, quality, and appropriateness (e.g., collinearity of variables, spatial bias of occurrence data) of input data^25^. However, mechanistic SDMs can be independent of the occurrence data because they are based on empirical parameterizations of biophysical processes^25^. This study selected CLIMEX, a prominent semi-mechanistic SDM developed by Sutherst and Maywald^26^, to project BPH distribution under various climate change scenarios. CLIMEX calculates the climatic suitability of the target organism based on climatic optimality and accumulated stresses and displays the year-round climate suitability of the target species as a single index, the Ecoclimatic Index. CLIMEX is a feasible approach for modeling the geographic distribution of BPHs, because they have been intensively studied to obtain sufficient core biophysical data. Furthermore, CLIMEX has the advantage of considering both the biophysical limitations of the target species and their actual geographical distribution patterns^27^. Thus, CLIMEX can recognize the actual boundaries of biophysical limitations by targeting proper occurrence data when fitting the parameters. To the best of our knowledge, no CLIMEX case study covers the full distribution of BPHs. Furthermore, there are limited applications of the CLIMEX model for modeling long-distance passive migratory species.

The objectives of this study were as follows: (1) to employ CLIMEX to model the geographic distribution of the overwintering area of BPHs, (2) to compare the overwintering CLIMEX model results with the conventional CLIMEX results, (3) to assess the impact of climate change to potentially cause a range shift of the overwintering area, and (4) to discuss how future BPH management strategies should be implemented by region. Additionally, a sensitivity analysis was performed to quantify the BPH response to each climatic factor and identify the parameters of functional importance to provide a greater understanding of the climatic factors that had the most impact on its species distribution.

## Materials and methods

### Current distribution and occurrence data of BPH

**Figure 1:**
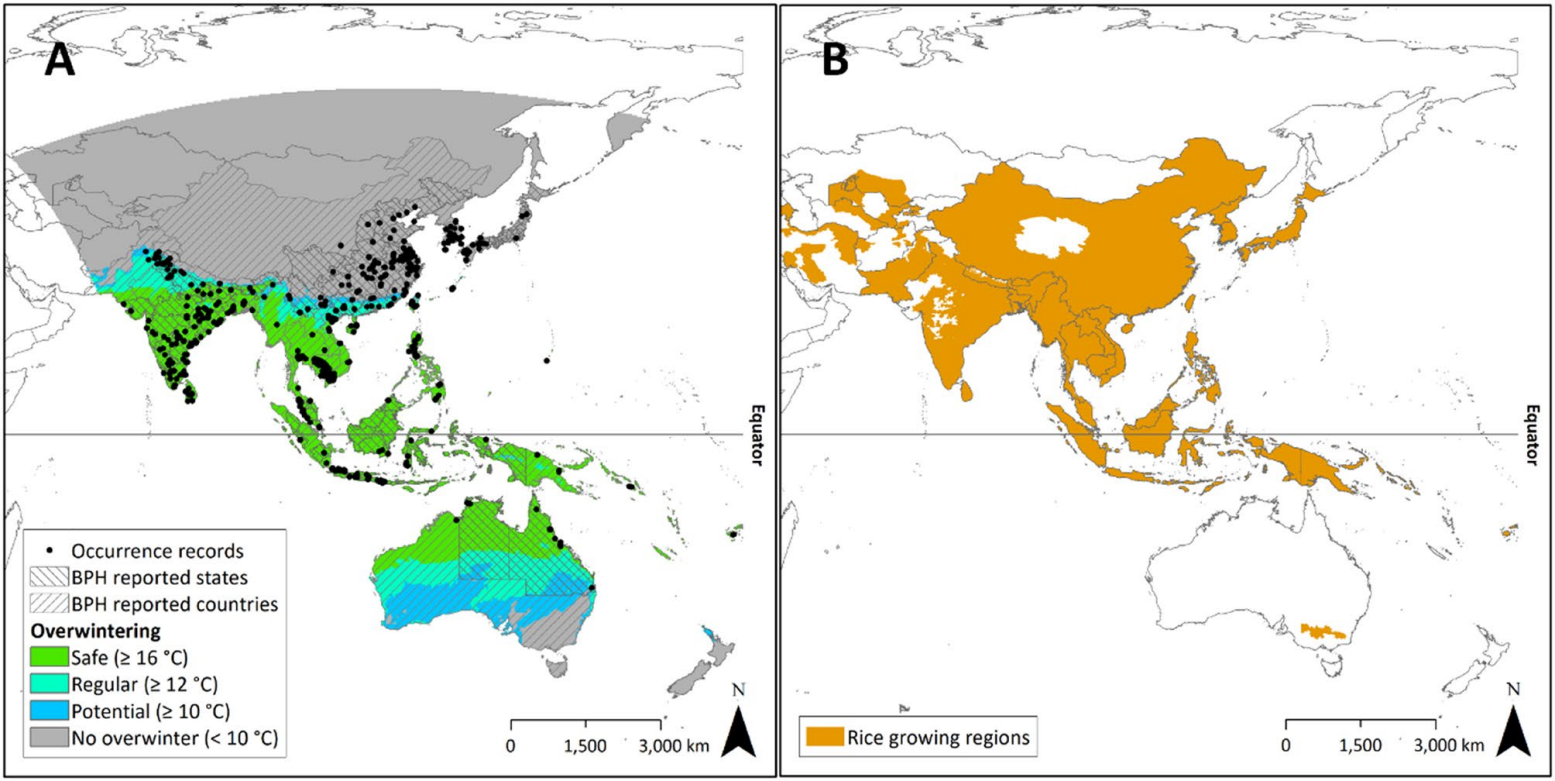
(**A**) The current distribution and overwintering boundary of *Nilaparvata lugens*. The locality data were collected from the literature and global databases (GBIF, and iNaturalist). Colors indicate to the class of overwinter: sky blue, turquoise, and green color indicate the coldest monthly winter temperature over 10, 12, and 16 °C, respectively. The cross-hatched area indicates the area where the distribution of *N. lugens* is reported (CABI/EPPO). (**B**) Rice growing regions in the RiceAtlas dataset.

A total of 706 occurrence records of BPH were obtained from published literatures and online databases^28^ (Fig. 1A). The countries and states where BPHs have been reported were obtained from the Centre for Agriculture and Bioscience International (CABI) Invasive Species Compendium and the European and Mediterranean Plant Protection Organization (EPPO) Global database^11^.

### CLIMEX model

The impact of future climate change on the climatic suitability for BPH was modeled to determine its potential geographical distribution using CLIMEX ver. 4.0.2 (Hearne Scientific Software, Australia). The initial model parameters were set using known biophysical information. The model was developed iteratively, whereby each parameter was individually adjusted to develop a model output that was closely aligned with the reported geographic distribution.

The Ecoclimatic Index (EI) was obtained from CLIMEX, and describes the overall climatic suitability of the target species based on comprehensive climatic and physiological data. The EI is an annual index of climatic suitability based on weekly calculations of the Annual Growth Index (GI_A_) and Annual Stress Index (SI_A_)^26^. The GI_A_ describes the combined temperature and moisture optimality for population growth scaled from 0 to 100 with a larger scaled number indicating a larger GI_A_. The SI_A_ describes the combined magnitude of climatic stresses (hot, cold, dry, and wet) scaled from 0 to 1000 with a larger scaled number indicating a larger SI_A_.

$$EI={GI}_{A}*{SI}_{A}$$

Detailed information of GI and SI components are described in Supplementary 1.

The EI was scaled from 0 to 100, where areas unsuitable for species development are indicated by 0. Only under year-round ideal conditions can the EI reach 100. Kriticos et al*.*^27^ explained that EI values can be classified into three arbitrary classes: unsuitable (0), moderate (1–29), and suitable (30–100). However, these classifications may be species-dependent and should be defined further in accordance with the actual occurrence of specific species in different regions. Because no previous study has classified the EI values of the BPH, the EI values in the overwintering areas were classified using a geometrical interval method in ArcGIS 10.8 software (Esri, Redlands, California, USA). The EI values are classified in five classes based on EI = 30, which is indicated as a threshold value for distinguishing suitable areas in the CLIMEX manual: not suitable (0), marginal (≤ 15), moderate (16–35), favorable (36–62), and very favorable (63 ≤). The average EI value for occurrences in the regular overwintering boundary (≥ 12 °C in the coldest month; detailed classes are explained in Section "Modeling strategy") is 29.8, indicating that the EI classification proposed in this study is reasonable.

The EI incorporates the effect of all climate stresses, and the stressor-specific results are presented in Supplementary 2–5. If any stress index exceeded 100, it was considered unsuitable. Because there is no specific rule for classifying stress indices below 100, this study categorized stress indices using an equal interval method: no stress (0), low (1–24), medium low (25–49), medium high (50–74), high (75–99), and uninhabitable (100).

### Climate data for current and climate change scenarios

The CLIMEX model requires the average monthly maximum and minimum temperatures, average precipitation, and relative humidity (RH) at 9:00 and 15:00^27^. For the current climate data, CliMond 10′ gridded global climate data (CM10_1975H_v1.2. mm) was used^29^. To represent the current period, the dataset was generated using climate data from 1960 to 1990. Future climate change data was generated based on the Representative Concentration Pathways (RCP) 2.6 and 8.5 climate change scenarios. Each RCP corresponds to different trends in future temperatures: RCP2.6, an optimistic scenario in which global temperature is expected to rise no more than 2 °C by 2100, and RCP8.5, a pessimistic scenario which could increase global temperatures by up to 5 °C by 2100^30,31^. The RCP scenario data from the Coordinated Regional Climate Downscaling Experiment (CORDEX) project were used to generate future input data^32^. The CORDEX project divides the Earth into 14 different domains and produces Regional Climate Model (RCM) results based on the General Circulation Model (GCM) outputs^33^. The CORDEX datasets are available from the Earth System Grid Federation (ESGF) data nodes.

To obtain appropriate climate datasets, this study searched for datasets that (1) covered the current geographic distribution of BPHs, (2) had the finest resolution, and (3) had all the climate variables needed to generate CLIMEX inputs. After comparing the known distribution of BPHs and CORDEX domains, this study selected three domains that cover the current geographic distribution of the BPH: East Asia (EAS), Southeast Asia (SEA), and Australasia (AUS). For these domains, three GCM datasets are available at a resolution of 0.22 decimal degrees: MOHC-HadGEM2-ES by the Met Office Hadley Centre (MOHC), MPI-M-MPI-ESM-LR by the Max-Planck-Institute for Meteorology (MPI-M), and NCC-NorESM1-M by the Norwegian Climate Center (NCC). All three GCM datasets were coupled with one RCM, REMO2015, from the Climate Service Centre, Germany (GERICS). Therefore, three GCM-RCM combinations (MOHC-HadGEM2-ES-REMO2015, MPI-M-MPI-ESM-LR-REMO2015, and NCC-NorESM1-M-REMO2015) were selected for this study. These datasets contain both RCP2.6 and RCP8.5 climate scenario data. Future projections for each RCP scenario are targeted at 2030 and 2080 to represent the near and far futures, respectively. A 30-year average was used to account for climate variability in each period: 2016–2045 for 2030 and 2066–2095 for 2080.

Because the CORDEX climate data did not include 9:00 and 15:00 h RH data, these were estimated using the method suggested by Kriticos et al*.*^29^. Temperature at 9:00 and 15:00 were estimated using the empirical relationship in the diurnal temperature range. The saturated vapor pressures at each temperature were then calculated using the Magnus equation, based on the empirical relationship using the dew point temperature^34^. In this step, the minimum temperatures are used as a proxy for the dew point temperature^34,35^ because it is assumed that the minimum temperature and dew point temperature tend to reach equilibrium at night^36^.

### Overwintering CLIMEX

#### Modeling strategy

The CLIMEX model may overestimate the biophysical limits of the BPH if the models are constructed using seasonal occurrence records from the entire known range because BPHs cannot overwinter in the temperate regions of China, Korea, and Japan. To overcome these constraints, the occurrence data were divided into two groups: those within and without the overwintering boundary. The first CLIMEX model was fitted to the occurrence data within the overwintering boundary (overwintering CLIMEX), and the second model was constructed using data from all regions where BPHs have been reported (conventional CLIMEX).

**Figure 2:**
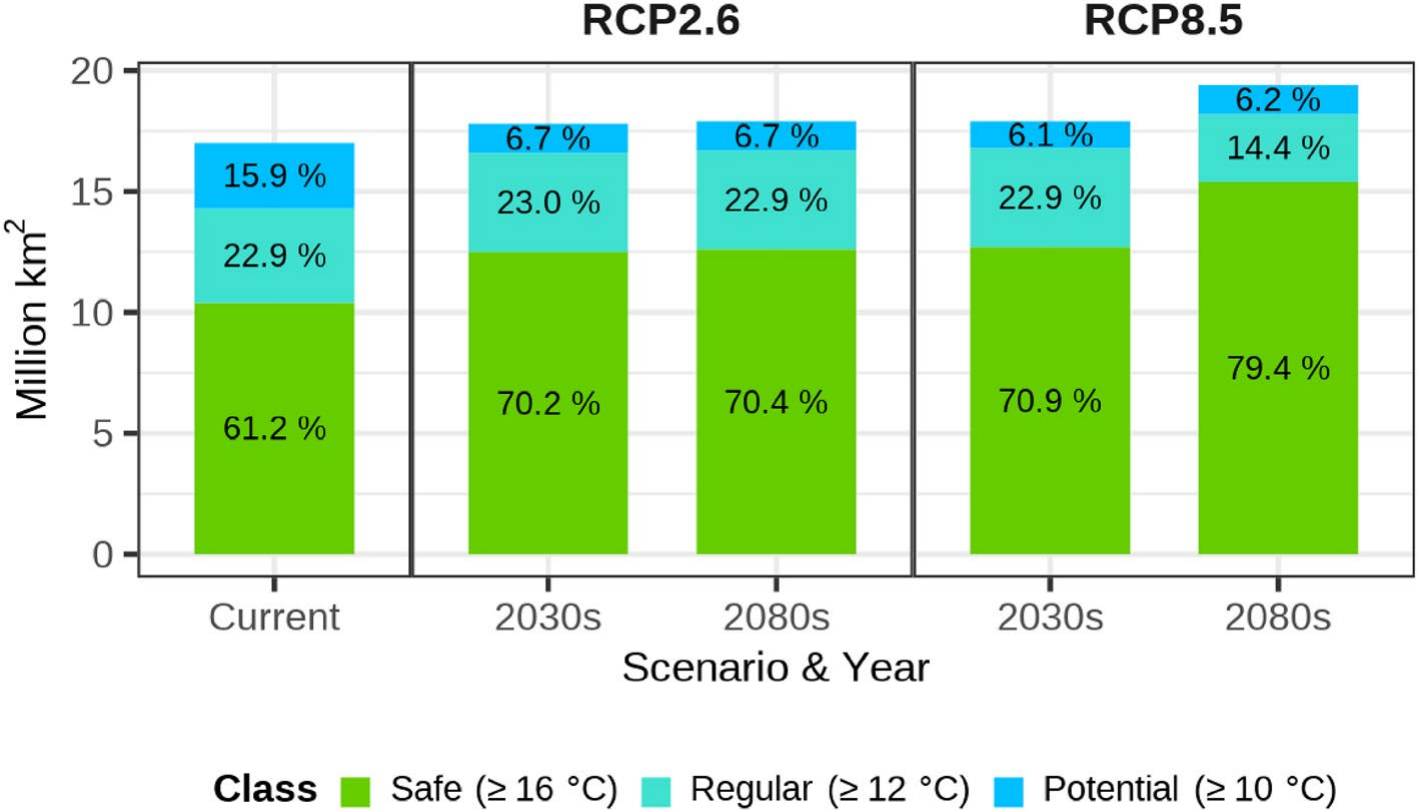
The area within the overwintering boundary of *Nilaparvata lugens* under the current and climate change scenarios. Colors indicate to the class of overwinter: sky blue, turquoise, and green color indicate the coldest monthly temperature over 10, 12, and 16 °C, respectively.

The overwintering boundary is divided into three classes based on the coldest monthly winter temperatures (T_coldest_): potential overwintering boundary (10 °C ≤ T_coldest_), regular overwintering boundary (12 °C ≤ T_coldest_), and safe overwintering boundary (16 °C ≤ T_coldest_) (see Fig. 1A for the current climate, and Supplementary 6 for the climate change). The changes in the area of each boundary are summarized in Fig. 2. These three classes were selected based on the following information. In general, January is the coldest month in the temperate regions of East Asia, including China^37^. Cheng et al*.*^15^ reported that the overwintering boundary of the BPH in China fluctuated between 21° and 25° N, which can be explained by using a 12 °C isotherm in January or a 2–3 °C extreme minimum temperature in winter. Moreover, BPH cannot survive when the mean January temperature is below 10 °C^38^, and thus January’s isotherm of 10 °C can be the index to determine the northern limit of the BPH^39^. Additionally, BPH can overwinter safely when the mean January temperature exceeds 16 °C^38^. Hu et al*.*^5^ used isotherms corresponding to average January temperatures of 10 and 16 °C as the intermittent, and constant overwintering boundaries, respectively. As the seasons are reversed in the Northern and Southern Hemispheres, this study used the mean temperature of the coldest month instead of the mean temperature of January to determine the overwintering boundary.

The model parameter fitting was conducted only within the rice growing regions because the BPH is monophagous on rice^11^. Rice growing regions were identified using RiceAtlas data^40^ (Fig. 1B).

#### Parameter fitting

##### Temperature

After the initial parameters were determined using relevant information and data from previous studies, the final parameters for fitting the model were selected by sequentially adjusting them to evaluate the potential geographical distribution of the BPH under the different climate change scenarios (Table 1). Because of the limited research on the biology of the BPH in the Southern Hemisphere, this study assumed that life history parameters from the Northern Hemisphere were similar to those in the Southern Hemisphere.

**Table 1:** Final CLIMEX parameter values fitted for *Nilaparvata lugens.*

Category	Code	CLIMEX parameter	Unit	Value
Growth Index (GI)				
Temperature Indices (TI)	DV0	Lower temperature limit	°C	11.6
	DV1	Lower optimal temperature	°C	26
	DV2	Upper optimal temperature	°C	30
	DV3	Upper temperature limit	°C	35
	PDD	Degree-day for complete generation	DD^a^	389
Moisture Indices (MI)	SM0	Lower moisture limit		0.18
	SM1	Lower optimal moisture		0.3
	SM2	Upper optimal moisture		1.5
	SM3	Upper moisture limit		2.5
Stress Index (SI)				
Cold Stress (CS)	TTCS	Cold stress temperature threshold (minimal temperature)	°C	0
	THCS	Cold stress temperature rate (minimal temperature)	°C/week	− 0.15
	DTCS	Cold stress degree-day threshold	DD	10
	DHCS	Cold stress degree-day rate	DD/week	− 0.003
Heat Stress (HS)	TTHS	Heat stress temperature threshold	°C	35
	THHS	Heat stress temperature rate	°C/week	0.0002
Dry Stress (DS)	SMDS	Dry stress threshold		0.25
	HDS	Dry stress rate	/week	− 0.008
Wet Stress (WS)	SMWS	Wet stress threshold, wet stress begins to accumulate		2.5
	HWS	When SM is above SMWS, Wet stress accumulates	/week	0.002

^a^Degree-day.

Values without units are dimensionless indices of soil moisture (0 is over-dry, 1 indicates 100% moisture content capacity).

The lower temperature limit (DV0) and the number of degree-days (DD) needed to complete one generation (PDD) were set to 11.6 °C and 389 DD, respectively^5^. The optimal temperature thresholds are reported as 28.2–29.8 °C for egg, 27.6–28.7 °C for nymphs, and 27.8–28.8 °C for egg to adult^41^. A recommended setting gap between the lower optimum (DV1) and upper optimum temperatures (DV2) is about 4 °C because the range between DV1 and DV2 is intended to cover 90–95% of the optimal growth^27^. Therefore, DV1 and DV2 were set to 26 °C and 30 °C, respectively.

The upper temperature limit (DV3) and heat stress temperature threshold (TTHS) are set to 35 °C. Piyaphongkul et al*.*^17^ reported that BPHs exhibited abnormal moving behavior due to heat stress above 34.9 °C. No egg and nymph could survive at 35 °C^41,42^. Based on the TTHS, the THHS rate was adjusted to 0.0002 after fine tuning using occurrence data from South Asia.

Cold stress (CS) determines whether a species can overcome extremely cold temperatures. The cold stress temperature threshold (TTCS) was fitted in an iterative manner from the default value of 2 °C in the CLIMEX Wet Tropical Template. Because the BPH is monophagous, the critical low temperature for rice plants could be closely linked to the critical low temperature for the BPH. According to Chen et al*.*^43^, the critical low temperature for the rice plant is between 0 and 2 °C while the supercooling point of BPHs (− 9.81 to − 7.6 °C) is lower than that of the rice plant (− 6.75 to − 2.04 °C). Therefore, the critical low temperature of the BPH was set to 0 °C for a conservative approach.

Other CS parameters, such as cold stress temperature rate (THCS), cold stress degree-day threshold (DTCS), and cold stress degree-day rate (DHCS), were fitted to predict the known overwintering boundary of BPHs in China. After iterative parameter fitting, THCS, DTCS, and DHCS were modified to − 0.15, 10, and − 0.003, respectively.

##### Moisture

Studies on the effects of moisture on BPHs under rice field conditions are limited. However, laboratory experiments have shown that BPHs prefer very humid conditions (RH > 90%)^44^, and are known to be distributed in almost every Southeast Asian and Oceanian country in humid tropical regions. Thus, the moisture index (MI), dry stress (DS), and wet stress (WS) parameters were initially adopted from the Wet tropical template in CLIMEX. The parameters were then fine-tuned using known BPH locality data. The Wet Tropical Template is a set of parameters that represents the species living in the most humid areas among the templates built in CLIMEX.

CLIMEX calculates weekly soil moisture values to estimate MI, SI, and WS. Although soil moisture may not directly affect the geographic distribution of insect pests, it can indirectly regulate their geographic distribution by limiting host plant availability. In the case of BPHs, drought stress alters the chemical composition of rice plants which results in reduced feeding preference^46^. In addition, laboratory experiments have indicated that the germination of rice seeds decreases rapidly at 30% soil moisture content, and no seeds germinate at 18% soil moisture content^46^. Thus, the low moisture limit (SM0) and dry stress threshold (SMDS) were both set to 0.18, and the lower optimal moisture level (SM1) was set to 0.3. The dry stress rate (HDS) parameter was set to -0.008 after the iterative fitting process to account for the BPH distribution in some dry regions of Pakistan and India.

It is difficult to find previous studies indicating that the distribution of BPH is limited by high moisture. Thus, other moisture-related parameters keep the default Wet Tropical Template values to describe the humid-tolerant nature of BPHs. These default parameters explain the distribution of tropical pests well^19^. Therefore, the upper optimal moisture (SM2), upper moisture limit (SM3), wet stress threshold (SMWS), and wet stress rate (HWS) were set to 1.5, 2.5, 2.5, and 0.002, respectively. The output results showed that these parameter values resulted in a good fit with the current distribution and reveal high EI values in the original focal areas.

### Conventional CLIMEX

A conventional model was constructed using the occurrence data from all areas where the BPH populations were observed for over a year. Because this conventional model was intended to compare the importance of applying an overwintering boundary in the fitting process, only the parameters related to cold stress were modified using an iteration method. Parameter values for this model were fitted using the northernmost distribution boundary in China, for example, the Hebei and Liaoning provinces. To make the conventional CLIMEX model categorize those regions into marginal levels (EI ≤ 15), all CS parameters (TTCS, THCS, DTCS, and DHCS) were modified in an iterative manner. Finally, the TTCS, THCS, DTCS, and DHCS values were set to − 15 °C, − 0.001, 0, and 0, respectively.

### Sensitivity analysis

Sensitivity analysis can identify the parameters for which a variation in its value will have the most significant impact on the model results^18,47^. A sensitivity analysis of the parameters of the overwintering CLIMEX model was conducted to quantify the response of BPHs using a built-in sensitivity analysis function to changes in temperature, soil moisture, and stress indices^27^. Because all the parameters in CLIMEX are not on the same scale, the default sensitivity test ranges were predetermined by a group of experts^27^. The sensitivity of each perturbation was evaluated based on the percentage change in three categories: EI, range, and core distribution change (Supplementary 7). Parameters with a greater effect on the model output are described as ‘sensitive,’ whereas those that have no impact on the model output are termed ‘insensitive.’

## Results

### Potential overwintering area of BPHs under the current climate

**Figure 3:**
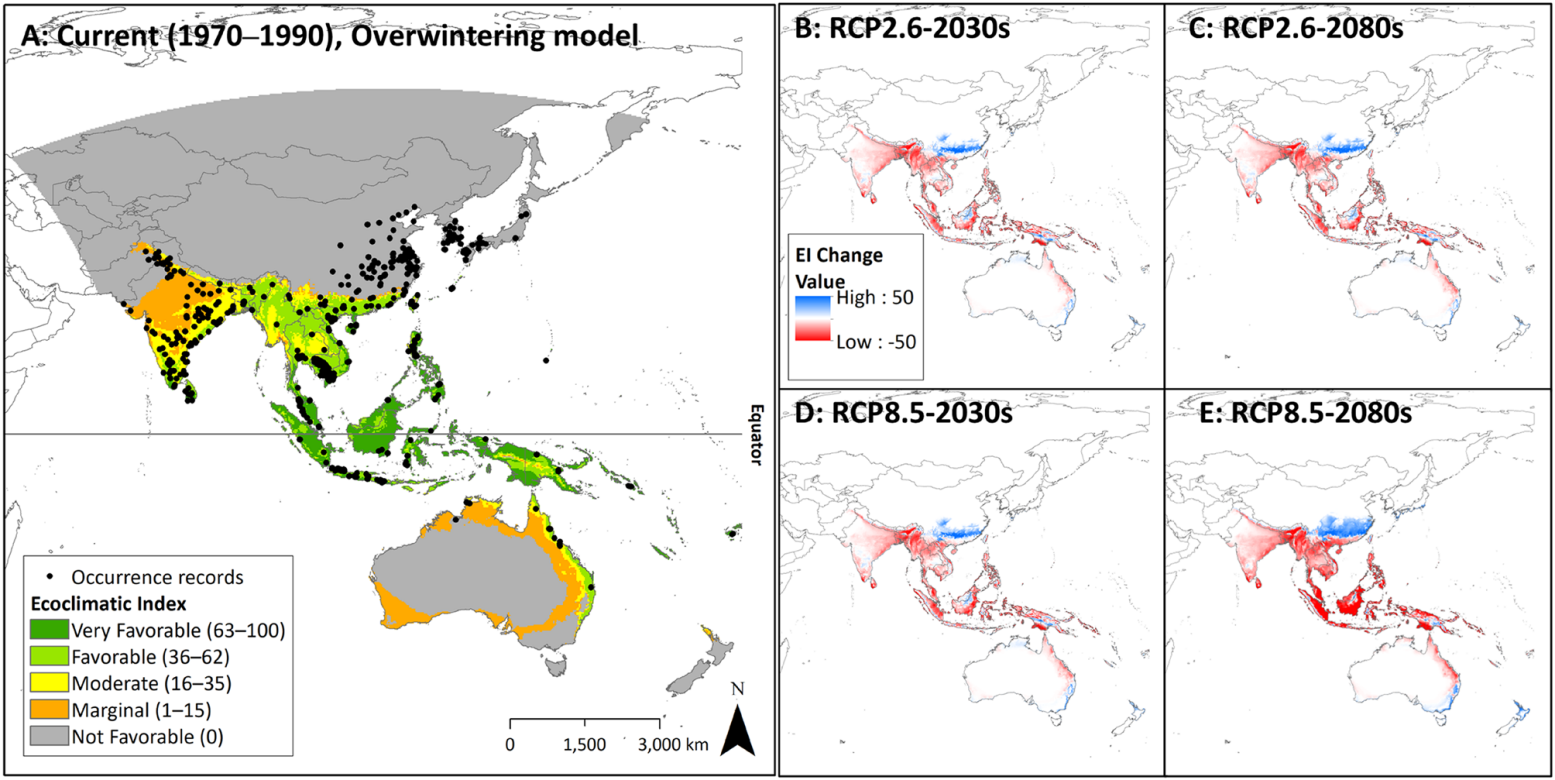
Estimated CLIMEX Ecoclimatic Suitability (EI) of *Nilaparvata lugens* under the current climate using the Overwintering CLIMEX model (**A**) and changes under climate change scenarios (**B**–**E**). Figure A explains overall climatic suitability of the overwintering area under the current climate conditions. The gray area indicates the location is not habitable from a long-term, year-round perspective. The orange, yellow, light-green, dark green area indicates ‘Marginal’, ‘Moderate’, ‘Favorable’, and ‘Very Favorable’ area. Figures **B** to **E** show the increase and decrease of EI. Red and blue indicate the decrease and the increase of EI, respectively.

The overwintering area under current climatic conditions was projected to determine how well the model fit the actual occurrence records. All overwintering records were located within the projected potential distribution (EI > 0), indicating a high consistency between the overwintering distribution and the modeling projection (Fig. 3A). The total habitable area (EI > 0) is estimated to fall within approximately 30% of the study domain (12.8 million km^2^ out of 41.1 million km^2^), of which marginal, moderate, favorable, and very favorable areas are relatively constant at 27.3, 18.6, 26.7, and 27.5%, respectively (Table 2). Very favorable, and favorable regions correspond to the safe overwintering boundary (Figs. 1A and 3A). The total area within the potential overwintering boundary from the study domain is 17.0 million km^2^, of which 61% is classified as a safe overwintering boundary (Fig. 2).

**Table 2:** Changes in area (million km^2^) according to five EI classes for *Nilaparvata lugens* under climate change scenarios.

Class	Current	RCP2.6		RCP8.5	
		2030s	2080s	2030s	2080s
Very favorable (63 ≤ EI)	3.4	1.5	1.4	1.3	0.4
	(27.5%)^a^	(12.7%)	(12.0%)	(11.2%)	(3.6%)
Favorable (36 ≤ EI < 63)	3.3	3.3	3.3	3.3	2.2
	(26.7%)	(27.3%)	(27.4%)	(27.5%)	(19.2%)
Moderate (16 ≤ EI < 36)	2.3	3.0	3.1	3.2	4.5
	(18.6%)	(24.6%)	(26.1%)	(26.9%)	(38.5%)
Marginal (1 ≤ EI < 16)	3.4	4.2	4.1	4.1	4.5
	(27.2%)	(35.4%)	(34.5%)	(34.4%)	(38.8%)
Habitable (Total) (0 < EI)	12.5	12.0	11.9	11.9	11.7

Total land area of study domain is about 41.1 million km^2^ (by merging CORDEX EAS, AUS, and SEA).

^a^The value in parenthesis indicates % area belonging to each EI class.

The total stress areas for HS, CS, DS, and WS were estimated to be 7.7, 23.7, 0.3, and 26.4 million km^2^, respectively (Table 3). When comparing them to the values estimated within the potential overwintering boundary, HS and WS areas overlap, but CS and DS decrease to 1.4, and 12.6 million km^2^, respectively. This suggests that the overwintering area is mainly controlled by CS, and that DS can be another important constraint in the expansion of the overwintering area under climate change.

**Table 3:** Changes in stress areas for *Nilaparvata lugens* under climate change scenarios.

Types		Current	RCP2.6		RCP8.5	
			2030s	2080s	2030s	2080s
Heat stress area (0 < HS)	Within total domain	7.74^b^	12.74	13.23	13.35	18.71
	Within potential overwintering boundary (10 °C ≤ T_coldest_^a^)	7.05	11.2	11.56	11.55	14.27
Cold stress area (0 < CS)	Within total domain	23.71	23.05	23.01	22.97	21.72
	Within potential overwintering boundary (10 °C ≤ T_coldest_)	1.43	0.74	0.71	0.77	0.12
Wet stress area (0 < WS)	Within total domain	0.26	1.51	1.5	1.54	1.65
	Within potential overwintering boundary (10 °C ≤ T_coldest_)	0.26	1.43	1.41	1.45	1.54
Dry stress area (0 < DS)	Within total domain	26.35	24.99	25.27	25.21	27.54
	Within potential overwintering boundary (10 °C ≤ T_coldest_)	12.57	14.22	14.41	14.28	15.25

Total land area of study domain is about 41.1 million km^2^ by merging CORDEX EAS, AUS, and SEA.

^a^Mean temperature of the coldest month.

^b^Area over the study domain (unit: million km^2^).

The northernmost overwintering area corresponds well to the previously reported northern limit, roughly along the latitude of 23° N in southern China (Fig. 3A). The northern limit of the habitable area (EI > 0) in the sub-Himalayan regions of Pakistan, India, and Nepal also corresponds to the potential overwintering boundary located between the latitudes of 23–25° N. The CS accumulated immediately in the areas above the potential overwintering boundary and was projected to be uninhabitable areas (CS = 100) (Supplementary 2A). These results suggest that CS accumulation due to harsh winter temperatures is the main constraint in most temperate regions such as northern China, Korea, and Japan.

Although the western parts of the Indochina peninsula, India, and Pakistan are in the safe overwintering boundary (Fig. 1A), EIs in these regions were classified mainly as marginal and moderate (Fig. 3A). Intense DS and mild HS were projected in these regions, which may have led to a decrease in EIs (Supplementary 3A and 4A). The south-central part of Pakistan, between latitudes of 26–30° N, is projected to have the highest HS among the study domains. To evaluate only the constraint of DS on the EIs, CLIMEX was re-run with a ‘temperature-only’ condition by excluding all the moisture-related indices (MI, DS, and WS). As WS was observed in very few areas of the projection (Table 3, Supplementary 5A), potential EI reductions by WS could be ignored in this test. The EIs observed in these regions changed from marginal to moderate, indicating that DS is a major contributor involved in the substantial reductions of EIs, even if the temperature conditions are favorable. (Supplementary 8).

Temperature conditions in most areas of the Southern Hemisphere were projected to be favorable for overwintering (Fig. 1). Northern Victoria and New South Wales, which are the only rice-growing regions in Australia, are projected to be non-overwintering regions due to CS (Supplementary 2A), and no BPH occurrence have been reported in these regions to date. Interestingly, BPH occurrence have been reported along the northern and eastern coasts of Australia, where wild *Oryza* species and alternative host plant species (*Leersia*) are distributed^48^. Until now, information on the life history and population dynamics of the BPH in these regions have been very limited; however, if the rice industry expands, these regions may become important habitats for focal populations. Most of the hot desert climate regions of Australia (e.g., the Northern Territory and Western Australia) are predicted to have severe HS and DS, suggesting that the current distribution in Australia could be limited by these two factors (Supplementary 3A and 4A).

Conventional CLIMEX projections suggest that the BPH can sustain populations throughout the temperate regions of East Asia (Supplementary 9). The northern overwintering boundary is projected further north to approximately 44° N (near Jilin Province, China). As a result, the conventional CLIMEX overestimates the habitable area (EI > 0) by 68%, compared to the overwintering CLIMEX, and most of the temperate regions where BPH cannot overwinter are classified as moderate or favorable (Supplementary 9). This overestimation is the result of blindly fitting the CLIMEX parameters to all known occurrence data. Unlike the overwintering model, the northern limits of the BPH habitats in migration areas seem to be determined by DS and the intensity of the low-level jet stream in summer, rather than by CS. These results highlight the importance of understanding the true limits of biophysical abilities when building a CLIMEX model for long-distance migratory organisms.

### Future potential distribution of BPHs with the overwintering CLIMEX

The future potential distributions of the BPH under two RCPs (2.6 and 8.5) and two different periods (2030s and 2080s) were projected (Fig. 3). The overwintering boundary remained similar to the current boundary in the combinations of RCP2.6-2030s and -2080s, and RCP8.5-2030s, but the boundary for RCP8.5-2080s significantly shifted northward to the latitude of mid-China (30° N near Hubei, Anhui, and Zhejiang), where the BPH cannot currently overwinter (Supplementary 6). Interestingly, no significant changes in the overwintering boundary were observed across the Himalayan mountainous regions. The total area within the potential overwintering boundary enlarged gradually across all combinations, ranging from 17.0 to 19.4 to million km^2^, and the proportion of the safe overwintering zone is greatest at RCP8.5-2080s (Fig. 2).

To evaluate the change in climatic suitability of overwintering areas, the EI values were compared to the combinations of RCPs and periods with the current values (Fig. 3). The total habitable area (EI > 0) remained relatively stable among all the combinations (11.7–12.0 million km^2^), but the EIs decreased as climate change progressed (Table 2). The proportion of very favorable and favorable areas decreased from 27.5 to 3.6%, and from 26.7 to 19.2% in RCP8.5-2080s, respectively. The opposite changes are projected in moderate and marginal.

As expected, the total CS area decrease gradually as climate change progresses (23.7–21.7 million km^2^). The CS within the potential overwintering boundary decrease substantially (1.4 at the current to 0.1 million km^2^ by RCP8.5-2080s), resulting in a shift in the overwintering boundary northward in China (Table 3 and Supplementary 2). In contrast, HS areas within the potential overwintering boundary increased substantially from 7.1 million km^2^ to 14.3 million km^2^ by RCP8.5-2080s (Supplementary 2).

**Figure 4:**
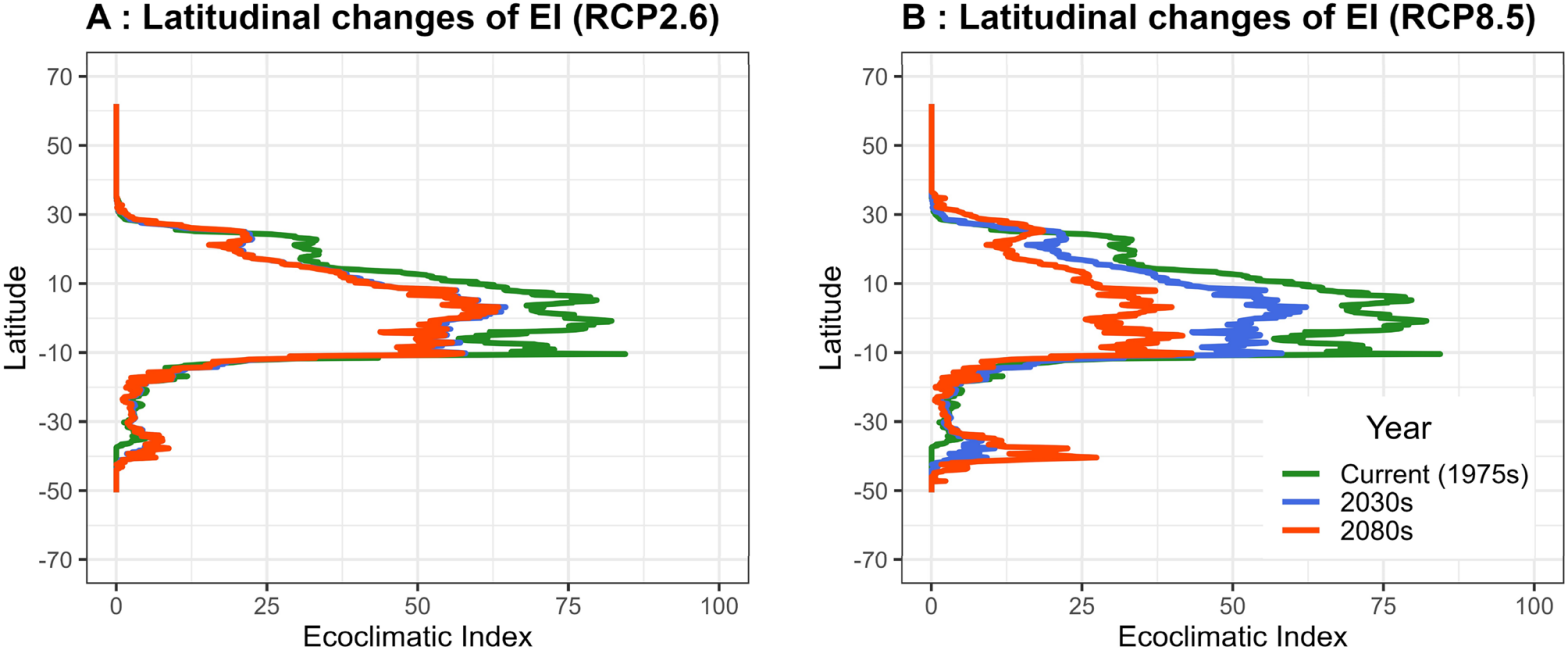
The latitudinal change of Ecoclimatic Suitability (EI) of *Nilaparvata lugens* under RCP2.6 (**A**), and RCP8.5 (**B**) climate change scenarios. Colors indicate periods: green, blue, and red indicates the current period (1960–1990), the 2030s, and the 2080s, respectively.

These maps show that climate change impacts on the suitability of BPH overwintering areas vary with latitude (Figs. 3 and 4). In general, the suitability peaks in the equatorial regions (10° N–10° S) and decreases in the high latitude regions. Furthermore, tropical regions are the most sensitive to climate change impacts, which EI decreases substantially over time. Interestingly, EI greatly enhanced over time in the mid-latitude regions in the southern hemisphere (30–40°S). The changes in the future potential distribution and area varied between regions (Fig. 3) and latitudinal changes are described in Fig. 4.

In China, the projections show that the potential distribution will extend to the north, and the favorable areas will become wider over time with climate change. marginal and moderate areas were layered at the northern boundary. These distribution expansions are closely linked to a decrease in CS in this region (Supplementary 2). Expansion of the overwintering area is mainly predicted in central and eastern China, but not in the western highland regions. Interestingly, Korea and Japan, where the BPH cannot currently overwinter, will increase in climatic suitability over time along their southernmost coasts, suggesting that the BPH could potentially overwinter and produce a focal population in these temperate areas in the future. However, a permanent establishment of BPH populations here is highly uncertain because overwintering populations have a low chance of surviving due to exposure to extreme short-term cold waves after rice field plowing.

In Australia, changes in the overwintering potential of major rice-growing areas under climate change are unclear, as CS is projected to decrease significantly, whereas HS and DS are projected to increase significantly. EIs in coastal areas of the Northern Territory and Queensland will decrease to marginal. Meanwhile, EIs in New South Wales and Victoria are improving as temperatures warm, allowing new overwintering populations to inhabit those areas. However, it is uncertain whether the BPH can easily survive under the natural conditions of these regions due to the significant increase in DS across those regions. Although North Island in New Zealand is projected to have suitable climatic conditions in the future, no economic damage is expected because only small trials of rice cultivation have been conducted in these areas^49^.

In South Asia, EI is projected to degrade because of climate change. Most of the current very favorable and favorable areas are degraded to marginal and moderate, respectively. The long and narrow habitable zone in the lower Himalayas is projected to become narrower and separated from mainland India owing to the increase in HS (Supplementary 3).

In the Indochina peninsula, EI in most of the region will decrease over time, while Vietnam is projected to remain favorable even under RCP8.5–2080. The low HS currently observed in western Myanmar becomes more intensified as climate change progresses, causing a sharp decrease in EIs from moderate to marginal.

The equatorial region experienced the most significant decrease in EI (Figs. 3 and 4). The current very favorable and favorable areas will decrease substantially over time, especially under RCP8.5. These decreases are probably due to the increased intensity of HS and DS with climate change (Supplementary 3 and 4). The mean EI values dropped to 50 in 2030 and 30 in 2080 under RCP8.5, compared to 70 at present.

Overall, the projected climatic suitability differed between regions. Most regions exhibited a decrease in suitability. Suitability in the equatorial region decreased in a latitude-dependent manner. However, its suitability in central and eastern China will greatly increase because of CS reduction over time.

### Parameter sensitivity analysis

The sensitivities of the EI, Range, and Core Distribution were evaluated in terms of percent change (Supplementary 7). The most sensitive parameter for EI Change is DV2 (5.0%), followed by SMDS (4.3%). For the Range and Core Distribution changes, the most sensitive parameter was SMDS (2.4 and 2.5%, respectively), followed by SM0 (0.9% for Range change), and DTCS and HDS (both 0.1% for Core Distribution change). Overall, the distribution and range of the BPH were very sensitive to DS, as indicated by the SMDS. The EI is most sensitive to the upper limit of temperature optimality (DV2), indicating that ongoing global warming will result in decreased EI as climate change progresses. Although DS was the most dominant and sensitive stress factor, HS showed the most significant increase as climate change progressed (Table 3). In addition, most of the HS areas were located within the potential overwintering boundary, whereas DS areas were relatively even (Table 3).

## Discussion

To the best of our knowledge, this is the first comprehensive study conducted at a regional scale to evaluate the impact of climate change on the biogeographical range shift of BPHs in relation to their overwintering areas. Although national-scale studies have been conducted, there were instances where the results did not align with this study. The overwintering boundaries in China shifted poleward, which is consistent with our projections^5,39^. However, unlike our projections which showed that the geographic distribution of BPH in the Indian region is shrinking owing to HS (Fig. 3), Guru-Pirasanna-Pandi et al*.*^50^ projected an expansion in the geographic distribution of BPHs in the Indian region. This discrepancy is probably caused by insufficient consideration of the geographic extent and climatic variables applied to the MaxEnt model. Data observed in the hottest and driest regions (e.g., Pakistan) were not included and only one temperature variable (annual mean temperature) was applied to the model.

This study clearly demonstrates that the conventional model-building process of CLIMEX is not appropriate for describing the climatic suitability of designated areas for long-distance migratory insects, such as BPHs. Because CLIMEX is designed to assess the year-round climatic suitability of the target species, the climatic suitability for BPHs will be overestimated if conventional CLIMEX is applied directly to BPHs. Therefore, to accurately evaluate the impact of climate change on BPH biogeographic distribution, understanding changes in climatic suitability within the overwintering boundary is a key priority. Although SDMs have been utilized to explain the geographical distribution of avian and mammalian migratory species^51,52^, few studies have applied SDMs to model the overwintering areas of passive migratory species. Hu et al*.*^6^ modeled the potential overwintering distribution of another important migratory planthopper, *Sogatella furcifera*, in Yunnan Province, China, with a focus on the influence of climatic and topographic factors. However, their study did not discuss the inherent limitations of SDMs in modeling the geographic distribution of passive migrators on a regional scale. To date, there are no known case studies that suggest significant advancements in modeling the biogeography of passive migratory insects.

This study found that changes in trends in overwintering boundaries and climatic suitability with climate change differs greatly between geographic regions. These changes have led to different statuses and differing importance of BPHs in different regions, indicating the need for a region/area-specific management strategy.

Areas close to the current overwintering boundary in China (20–30° N) should be intensively monitored for how the boundary shifts to higher latitudes during the course of climate change. Managing this region is important because China is the world’s most intensive rice production region, producing more than 11 million tons per year^40^. It also provides a major source population of BPHs that migrate to temperate zones. Therefore, the northward expansion of BPHs in China is a significant threat to future food security in East Asia. Overwintering sites should be properly managed to reduce the risk posed by BPHs in these regions. To achieve this, rice stubble, which can serve as an overwintering site^53^, should be removed after rice harvest to reduce the overwintering BPH density and carefully apply the ratooning cultivation method in this region. Ratoon rice is grown from the seedlings that remain at the nodes of the rice stubble following the harvest of the main rice crop^54,55^. Although ratoon rice has economic and environmental advantages^56^, it is useful in avoiding a high overwintering density in this region^57^.

Temperate regions, including northern China, Korea, and Japan, should focus on how the future density and timing of the first mass migration will affect the establishment of these regions. Future BPH risk will be closely associated with the northward shift of the overwintering boundary and changes in the East Asian jet stream (EAJS) with climate change. Climate change is expected to alter the patterns of the low-level jet streams that deliver BPH populations to temperate East Asia^14,58^. Xin et al*.*^59^ projected that the EAJS could weaken under climate change, but the jet core (the region of the jet stream axis with the greatest winds) is expected to shift northward. Therefore, when the overwintering boundary of the BPHs follow the EAJS well, the BPH potentially migrates to temperate zones earlier and more frequently than it currently does. Unfortunately, a comprehensive understanding of the future EAJS and migration ecology of BPH remains challenging.

Newly developed rice-growing areas in Australia should prevent the spread of focal BPH populations. Recently, multiple rice industry bodies have begun to develop rice-growing centers in northern and northeastern Australia^60,61^. As the occurrence of BPHs has already been reported in the Northern Territory and Queensland^48,62^, outbreaks of BPHs will be possible in these regions if a large rice production complex is established. Because limited information is available on the ecology of the Australian BPH population, continuous monitoring and further studies are urgently required. In particular, host switching is an important topic for the Australian population because most BPHs in Australia are found in alternative hosts, such as *Leersia* and wild rice^48^. According to Heinrichs and Medrano^63^, BPH nymphs bred on *Leersia hexandra* showed higher mortality than those bred on rice. However, the survival of the F_1_ progeny improved when bred with rice-eating BPHs. Thus, how the *Leersia*-eating population adapts to rice plants is a key topic in understanding the future damage of Australian BPH populations.

The Indochina Peninsula and South Asia, located between 20° S and 20° N should focus more on rice productivity rather than BPH risk. The risk of BPH infestation may be reduced in the future owing to increased heat stress in these regions. In the purview of biosecurity, the degrading EI of BPHs will change positively. However, rice is also vulnerable to increasing heat stress caused by climate change, especially during the flowering stage resulting in imperfect seedling emergence and rooting, reduced tillering, sterility, and reduced grain filling^64–66^. Thus, avoiding or reducing high-temperature stress in rice plants (e.g., cultivating heat-tolerant rice varieties and shifting planting dates) will be the most important climate change adaptation strategy in the future.

Although CLIMEX is a widely used species distribution model, it has some limitations that need to be considered in future research. The CLIMEX model developed in this study was mostly based on atmospheric temperatures and did not consider the microclimate of the paddy fields. The microclimate of paddy fields is highly dependent on agricultural practices, especially irrigation and flooding during rice cultivation^67^. The daily peak temperature under the rice canopy can be substantially reduced by the cooling effect^67–69^. Therefore, if microclimate data according to various rice cultivation practices can be included in the CLIMEX model, a more accurate and reliable geographic distribution of BPHs can be predicted with climate change.

Given that the BPH is monophagous, the findings of this study need to be considered alongside future projections of rice area. From 1997 to 2016, global rice production rose from 577 million tonnes to 753 million tonnes, and the cultivated area increased from 161.7 million hectares in 2010 to 165.04 million hectares in 2022^70–72^. Similarly, in China, where significant changes in BPH overwintering areas are anticipated, rice areas have stabilized at around 30 million hectares, with rice production quadrupling between 1949 and 2015^73^. Meanwhile, the rice-growing area has moved northwards, with double-cropped rice decreasing in southwestern China and single-cropped rice significantly increasing in northeastern China^73^. These shifts are expected to accelerate with climate change, as they are part of adaptation strategies. According to Zhang et al.^74^, the expansion of suitable areas for rice in northern China will intensify under the RCP8.5 compared to the RCP2.6, alongside a reduction in suitable areas for cultivation in some southern parts of China. Interestingly, these changes have similar trends to changes in the suitability of BPH overwintering areas predicted in this study. Therefore, it will be important how well the northward expansion of the BPH overwintering area aligns with the northward shift of the rice cultivation area. However, the shift in rice areas is influenced not only by climate but also by socio-economic factors. Thus, to predict future BPH damage, it is crucial to consider changes in rice-growing areas resulting from climate change and socio-economic factors.

Despite these limitations, this study clearly demonstrates the usefulness of modeling the distribution of the overwintering area of a migratory pest using CLIMEX. In future studies, a comprehensive climate change impact assessment that integrates range shifts of overwintering areas and migration ecology is required. In regions where BPH migrates long distances and settles, a short-term decision-making system that assesses the risk of initial mass migration is required. As weekly GIs have been utilized to assess the short-term dynamics of insect pests^75,76^, the risk from migratory populations in temperate regions can be assessed by analyzing weekly GIs. Interestingly, we found that weekly GIs during the rice-growing season in temperate regions had similar intensities to those in the overwintering regions, suggesting that the CLIMEX model parameters developed in this study (fitted to overwintering areas) can potentially be used to evaluate migration areas as well (Supplementary 10). To refine this approach, combining it with migration models is expected to enable more accurate predictions of potential BPH damage. For planthoppers, several forward and backward trajectory models have been developed^77,78^. The CLIMEX model presented in this study focuses on predicting the distribution of overwintering areas, so integrating it with forward-type trajectory models will be able to simulate changes in northward migratory populations after overwintering. Hence, integrating migration models with SDMs will greatly advance the development of management strategies for passive migratory insects like BPH. We believe that this study will contribute to the establishment of management plans for BPH under climate change.

## Conclusion

In establishing a management plan for migratory pests, the top priority is to clarify the distribution of the major source habitats. The results of the present study suggest that neglecting the biophysical limitations of migratory insect pests may lead to a serious overestimation of their geographic distribution. This study also showed that there would be a range shift in the overwintering areas of BPH under climate change. Ongoing climate change may increase the risk posed by BPH in temperate regions because increasing winter temperatures will expand their overwintering boundaries. However, the benefit of expanding toward temperate regions may be offset by the retraction of overwintering areas in tropical to sub-tropical regions caused by increasing heat stress. Given that shifted overwintering areas alter the magnitude of migratory populations, it is crucial to establish a regional management strategy for each area to adapt to climate change. This study underscores the significance of considering biophysical limitations in CLIMEX studies to accurately estimate the source habitats of migratory pests.

### Supplementary Information

Supplementary Information.

## Data Availability

The study data are available from the corresponding author on reasonable request.
