# Supplementary material for: Potential range shift of a long-distance migratory rice pest, Nilaparvata lugens, under climate change
Source: Sci Rep. 2024 May 21;14:11531. doi: 10.1038/s41598-024-62266-x (PMC11109201; doi:10.1038/s41598-024-62266-x)
Supplement: Supplementary file 1 — Supplementary Information. [file 41598_2024_62266_MOESM1_ESM.docx]

**Supplementary 1**. Detailed explanation of Growth Index and Stress Index.

The GI_A_ describes the potential for population growth scaled between 0 and 100 (a higher value means a better condition) and is a standardized mean of Weekly Growth Index (GI_W_). GI_W_ describes the potential weekly population growth of the target species when climatic conditions are favorable, obtained by multiplying weekly Temperature Index (TI_W_) and weekly Moisture Index (MI_W_).

$${GI}_{A}=100\sum_{i=1}^{52} {GI}_{Wi}/52$$

$${GI}_{W}={TI}_{W}*{MI}_{W}$$

The SI_A_ limits survival during the unfavorable season and determines the boundary of a species' geographic distribution. It is scaled between 0 to 100, and a higher value means a worse condition. The SI_A_ consists of cold (CS_A_), heat (HS_A_), dry (DS_A_), and wet individual stress (WS_A_) indices. The weekly stresses for CS, HS, DS, and WS accumulate only when the climate condition exceeds user-defined threshold values. When the weekly stress occurs continuously, it is added up until the stress does not occur. Individual stress indices range from 0 to 1000, and a greater value means more severe stress. When individual stress indices exceed 100, they are corrected to 100 and finally have a value from 0 to 100. That is, greater value means greater stress theoretically, but no more additional impacts on the SI_A_ over 100. Thus, if a single stress is greater than 100, the target species cannot inhabit that location as the final SI_A_ drops to 0. After computing all individual stress indices, all are combined in the SI_A_ as below.

$${SI}_{A}=\left( 1-\frac{{CS}_{A}}{100} \right)*\left( 1-\frac{{HS}_{A}}{100} \right)*\left( 1-\frac{{DS}_{A}}{100} \right)*\left( 1-\frac{{WS}_{A}}{100} \right)$$

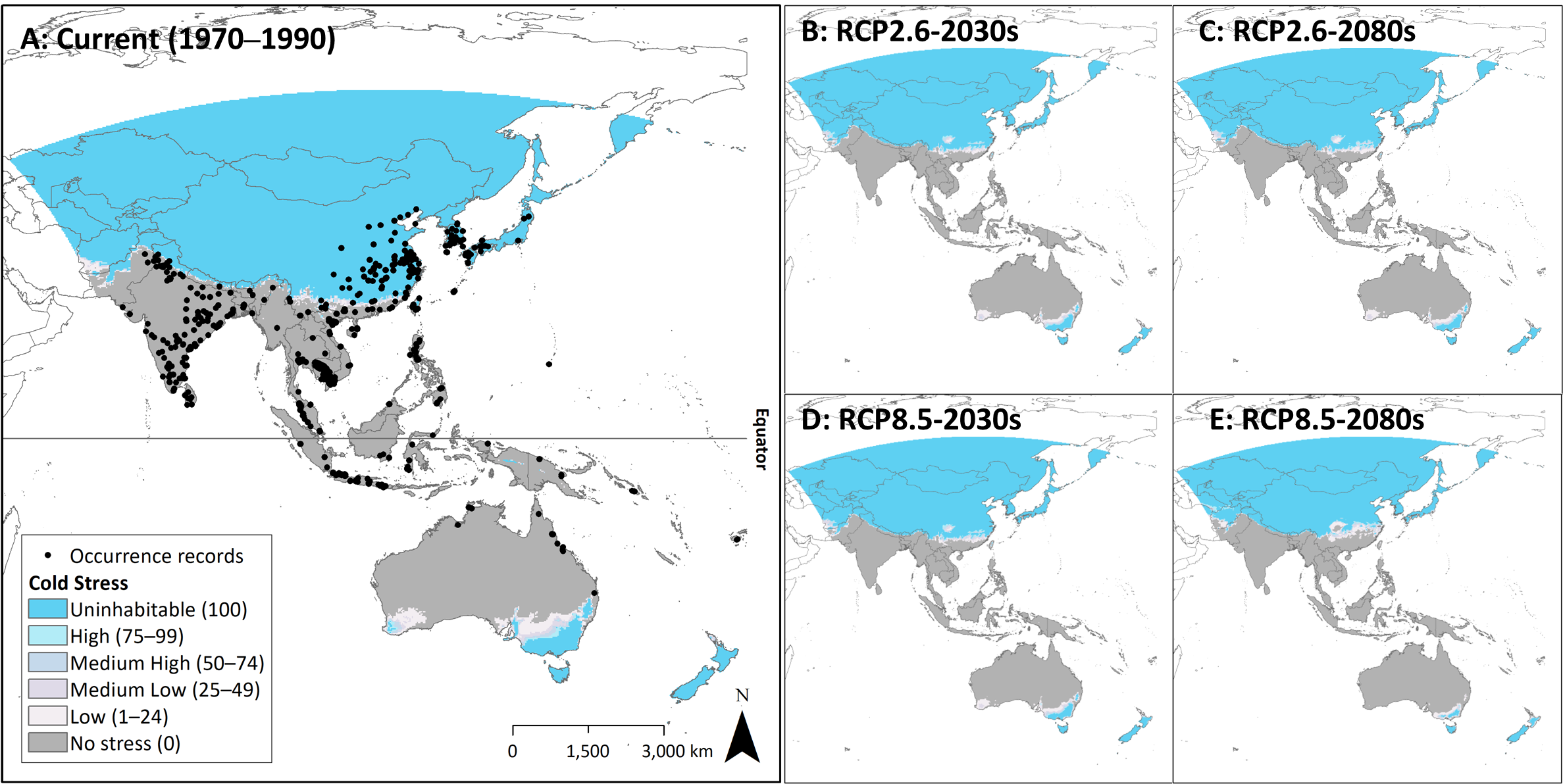


Supplementary 2. Estimated CLIMEX Cold Stress (CS) of *Nilaparvata lugens* under the current climate (A), RCP 2.6 climate change scenario (B and C), RCP 8.5 climate change scenario (D and E). The gray area indicates that no CS accumulated over the year and the blueness indicates the severity of the cold stress. If stress achieve 100, BPH cannot survive at the location. Values between 1 to 99 were classified using the equal interval method to display the severity.


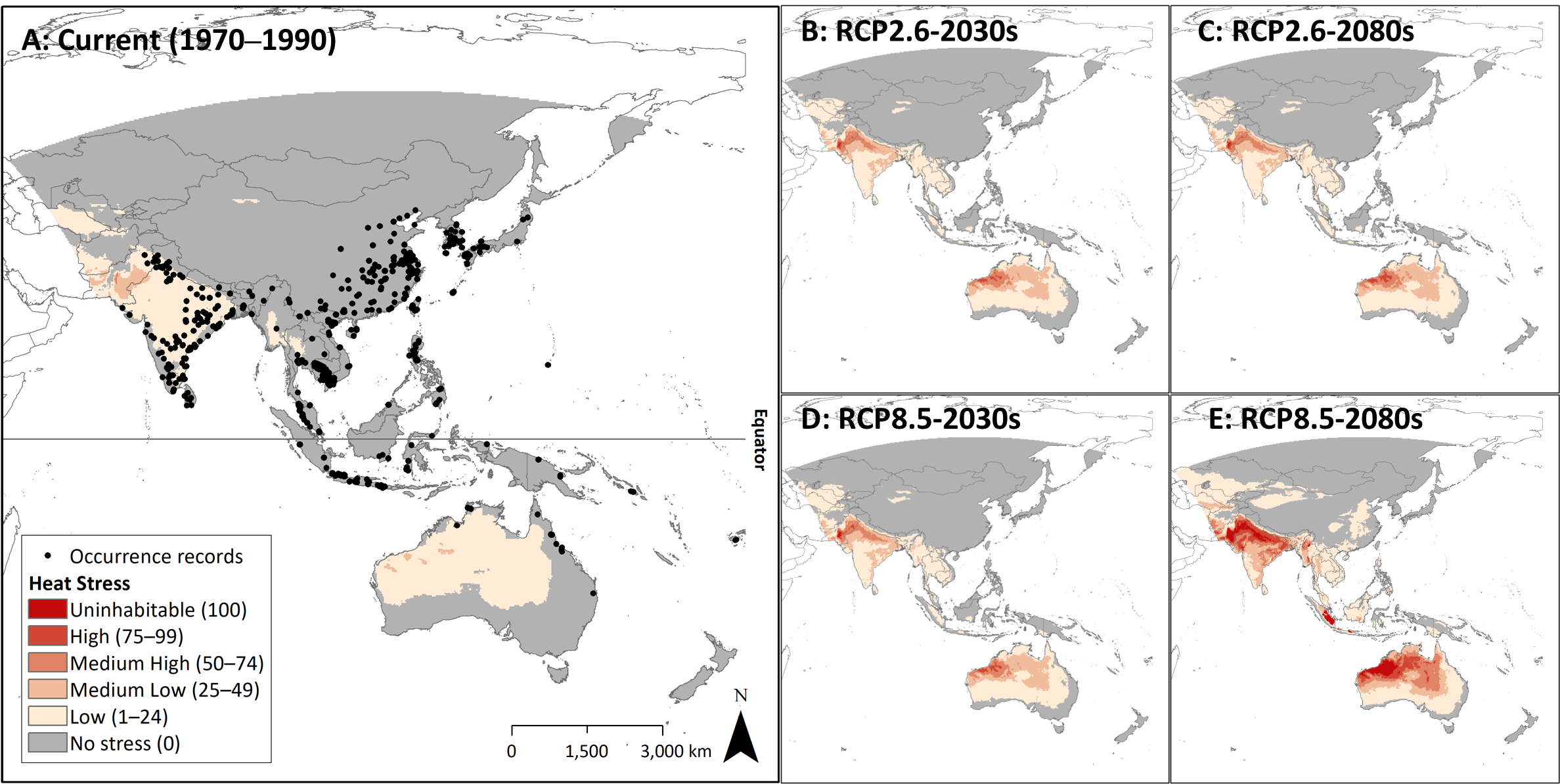


Supplementary 3. Estimated CLIMEX Heat Stress (HS) of *Nilaparvata lugens* under the current climate (A), RCP 2.6 climate change scenario (B and C), RCP 8.5 climate change scenario (D and E). The gray area indicates that no HS accumulated over the year and the redness indicates the severity of the heat stress. If stress achieve 100, BPH cannot survive at the location. Values between 1 to 99 were classified using the equal interval method to display the severity.


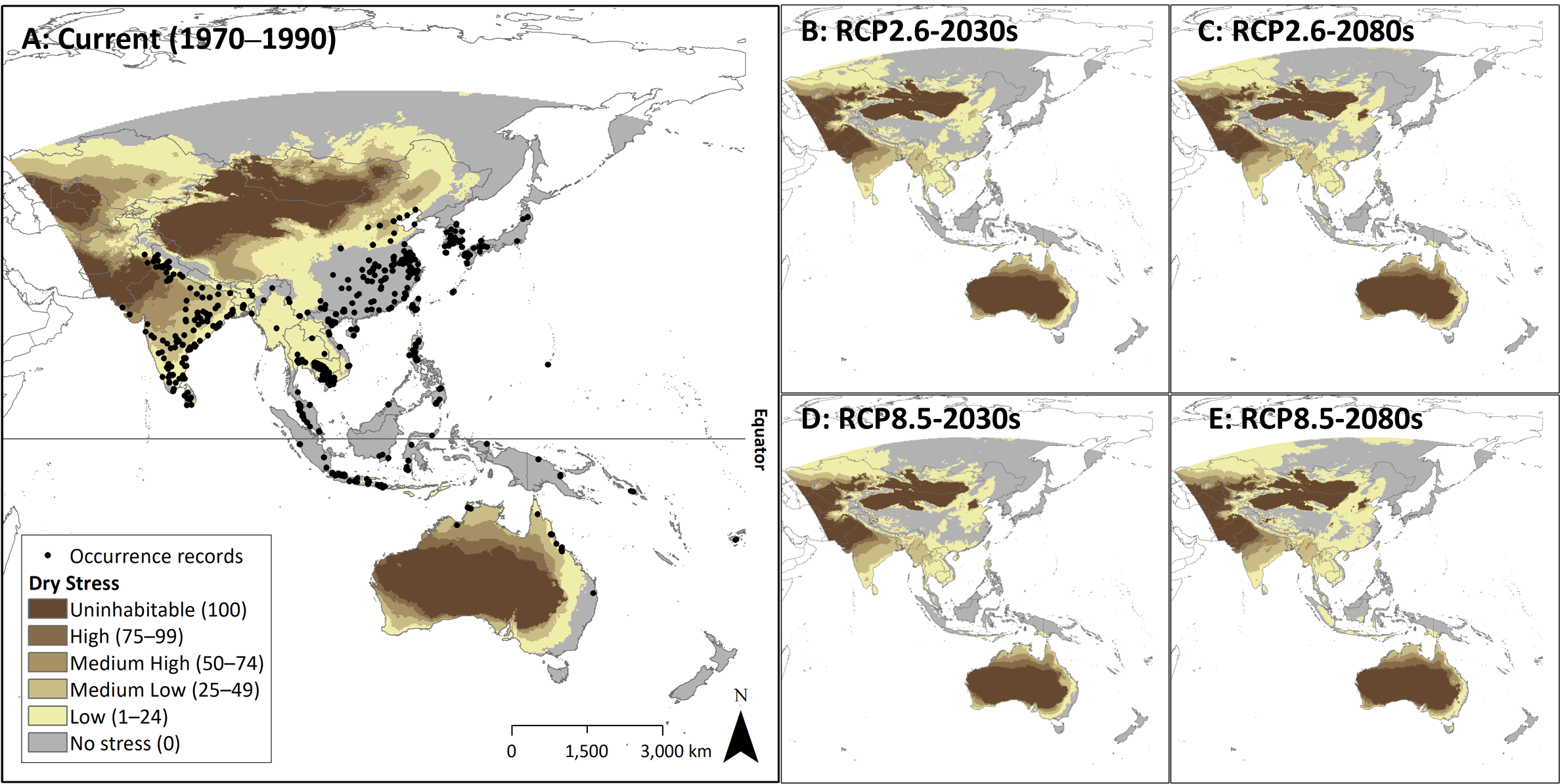


Supplementary 4. Estimated CLIMEX Dry Stress (DS) of *Nilaparvata lugens* under the current climate (A), RCP 2.6 climate change scenario (B and C), RCP 8.5 climate change scenario (D and E). The gray area indicates that no HS accumulated over the year and the brownness indicates the severity of the dry stress. If stress achieve 100, BPH cannot survive at the location. Values between 1 to 99 were classified using the equal interval method to display the severity.


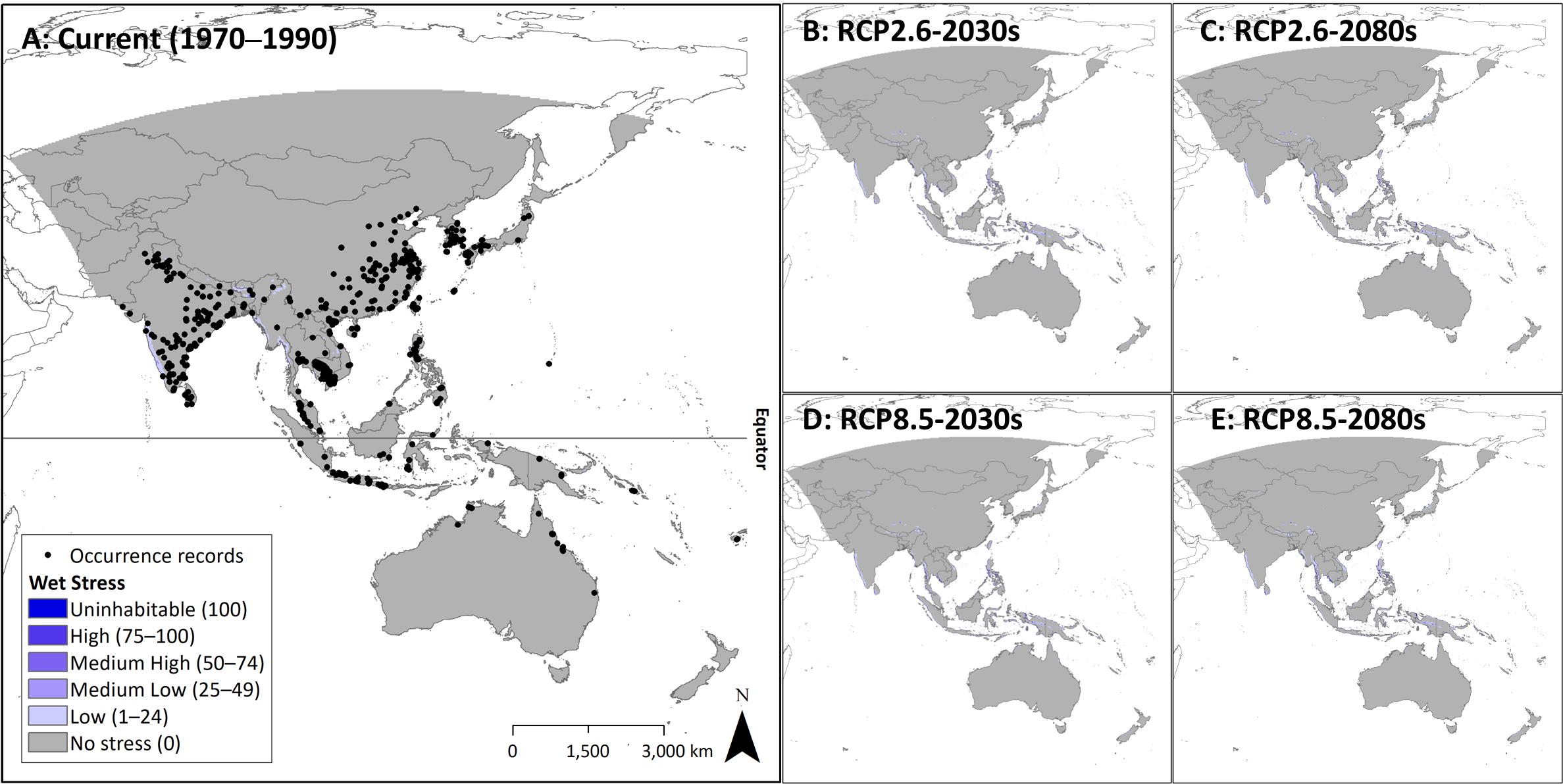


Supplementary 5. Estimated CLIMEX Wet Stress (WS) of *Nilaparvata lugens* under the current climate (A), RCP 2.6 climate change scenario (B and C), RCP 8.5 climate change scenario (D and E). The gray area indicates that no HS accumulated over the year and the blueness indicates the severity of the heat stress. If stress achieve 100, BPH cannot survive at the location. Values between 1 to 99 were classified using the equal interval method to display the severity.


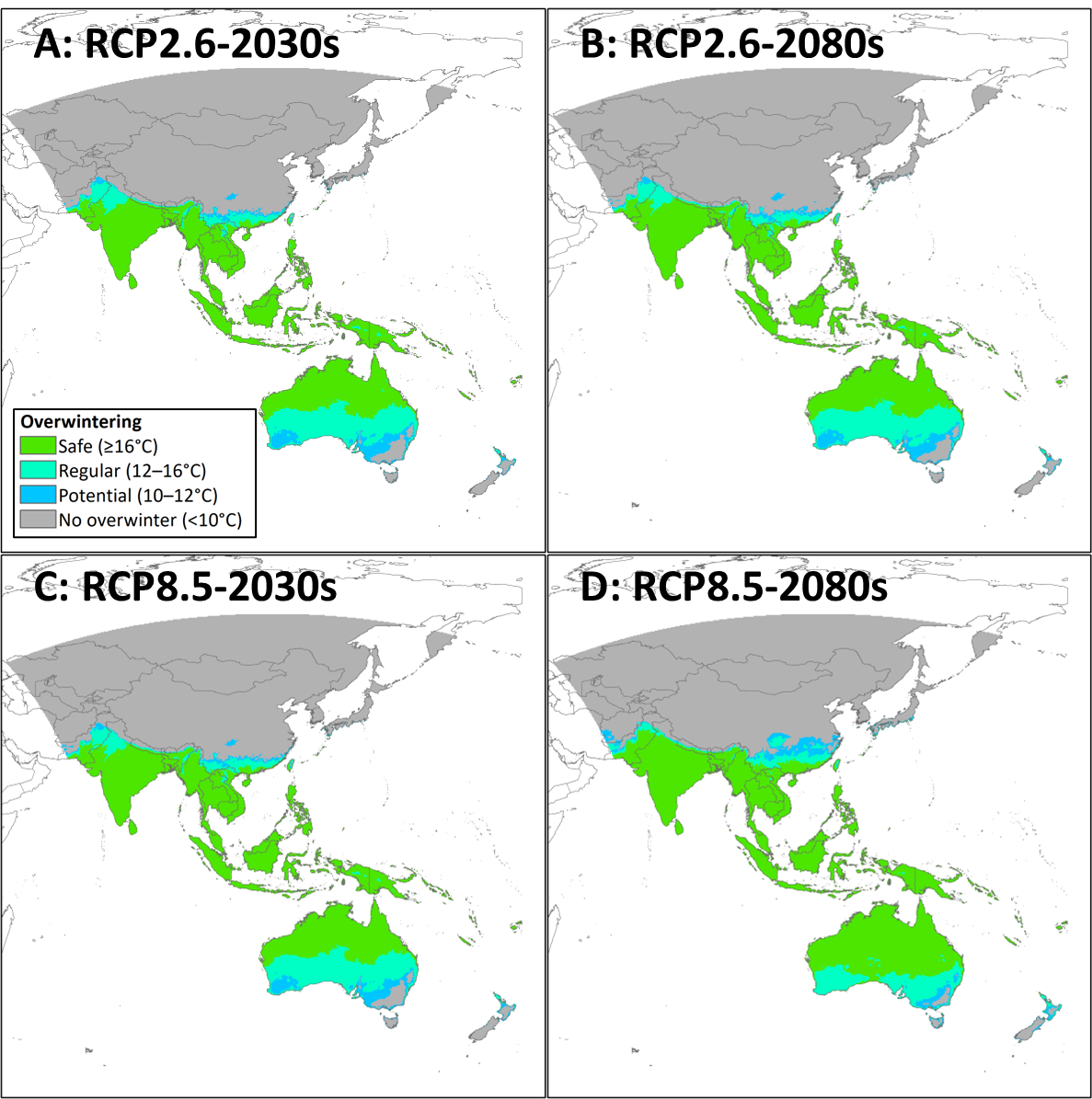


Supplementary 6. Overwintering area of *Nilaparvata lugens* under RCP 2.6 (A and B) and RCP 8.5 (C and D) climate change scenarios. Sky blue, turquoise, and green color indicate the coldest monthly temperature over 10, 12, and 16 °C, respectively.

Supplementary 7. CLIMEX sensitivity analysis results for *Nilaparvata lugens*.

| **Parameter** | | **Test Range** | | | **Sensitivity** | | |
| --- | --- | --- | --- | --- | --- | --- | --- |
|  |  | **Low** | **Default** | **High** | **EI Change^a^** | **Range Change^b^** | **CD Change^c^** |
| Limiting low temperature | DV0 | 10.6 | 11.6 | 12.6 | 0.9 | 0.1 | 0 |
| Lower optimal temperature | DV1 | 25 | 26 | 27 | 2.1 | 0 | 0 |
| Upper optimal temperature | DV2 | 29 | 30 | 31 | **5.0** | 0.1 | 0 |
| Limiting high temperature | DV3 | 34 | 35 | 36 | 2.2 | 0.6 | 0 |
| Limiting low moisture | SM0 | 0.08 | 0.18 | 0.28 | 2.1 | 0.9 | 0 |
| Lower optimal moisture | SM1 | 0.2 | 0.3 | 0.4 | 1.9 | 0.5 | 0 |
| Upper optimal moisture | SM2 | 1.4 | 1.5 | 1.6 | 2.2 | 0 | 0 |
| Limiting high moisture | SM3 | 2.4 | 2.5 | 2.6 | 0.7 | 0 | 0 |
| Cold Stress Temperature Threshold | TTCS | -1 | 0 | 1 | 0.3 | 0 | 0 |
| Cold Stress Temperature Rate | THCS | -0.18 | -0.15 | -0.12 | 0 | 0 | 0 |
| Cold Stress Degree-day Threshold | DTCS | 9 | 10 | 11 | 1.2 | 0.4 | 0.1 |
| Cold Stress Degree-day Rate | DHCS | -0.0036 | -0.003 | -0.0024 | 0.7 | 0.2 | 0 |
| Heat Stress Temperature Threshold | TTHS | 34 | 35 | 36 | 0.2 | 0 | 0 |
| Heat Stress Temperature Rate | THHS | 0.00016 | 0.0002 | 0.00024 | 0.1 | 0 | 0 |
| Dry Stress Threshold | SMDS | 0.15 | 0.25 | 0.35 | 4.3 | **2.4** | **2.5** |
| Dry Stress Rate | HDS | -0.012 | -0.01 | -0.008 | 1.0 | 0.4 | 0.1 |
| Wet Stress Threshold | SMWS | 2.4 | 2.5 | 2.6 | 0.1 | 0 | 0 |
| Wet Stress Rate | HWS | 0.0016 | 0.002 | 0.0024 | 0.1 | 0 | 0 |
| Degree-days per Generation | PDD | 311.2 | 389 | 466.8 | 0 | 0 | 0 |
| ^a^Percent change of the Ecoclimatic Index: The sensitivity of the overall climatic suitability ^b^Percent change of the Range: Locations that EI is 1 or more ^c^Percent change of the Core Distribution: Locations that are warm enough to complete at least one generation, and do not accumulate any stress Values in bold are the most sensitive parameters in each test | | | | | | | |


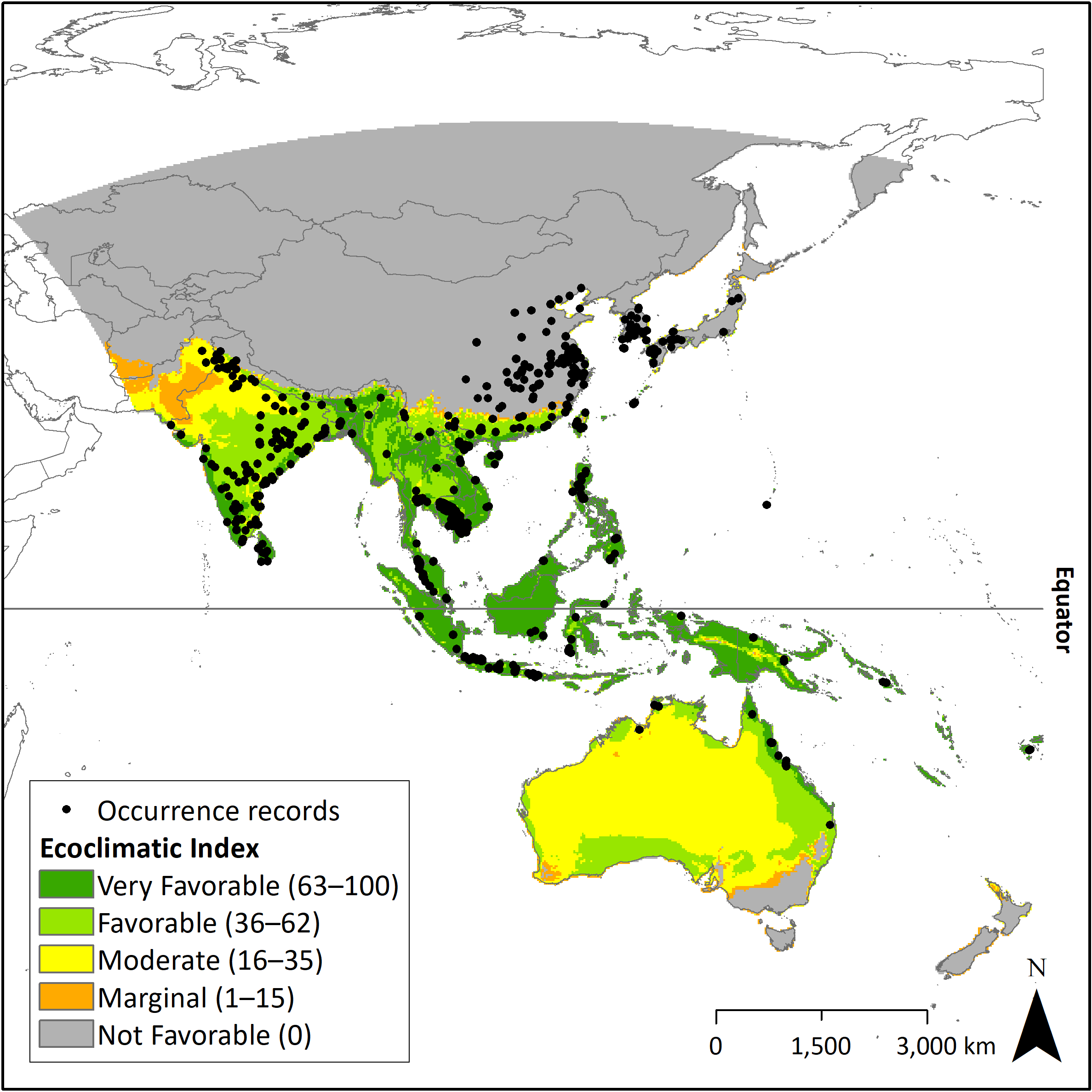


Supplementary 8. Projected temperature-only CLIMEX Ecoclimatic Suitability (EI) of *Nilaparvata lugens* under the current climate. The EI values explain overall climatic suitability of the overwintering habitat of *N. lugens*. The gray area indicates the location is not habitable from a long-term, year-round perspective.


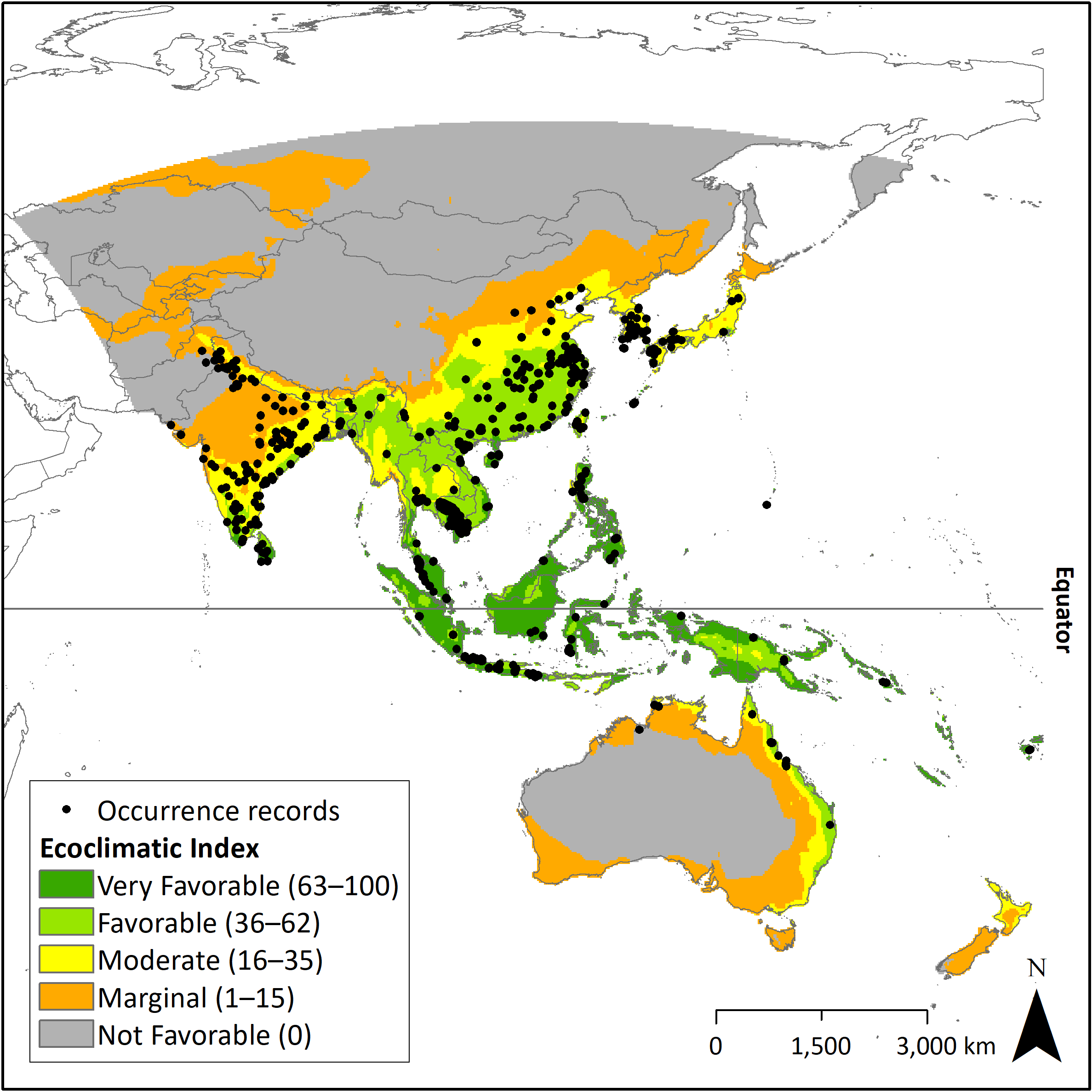


Supplementary 9. Estimated CLIMEX Ecoclimatic Suitability (EI) of *Nilaparvata lugens* under the current climate using the Conventional CLIMEX model. This model is fitted to the all-known occurrence records of *N. lugens* whether the occurrences are available to overwinter or not. The gray area indicates the location is not habitable from a long-term, year-round perspective. The orange, yellow, light-green, dark green area indicates ‘Marginal’, ‘Moderate’, ‘Favorable’, ‘Very Favorable’ area.


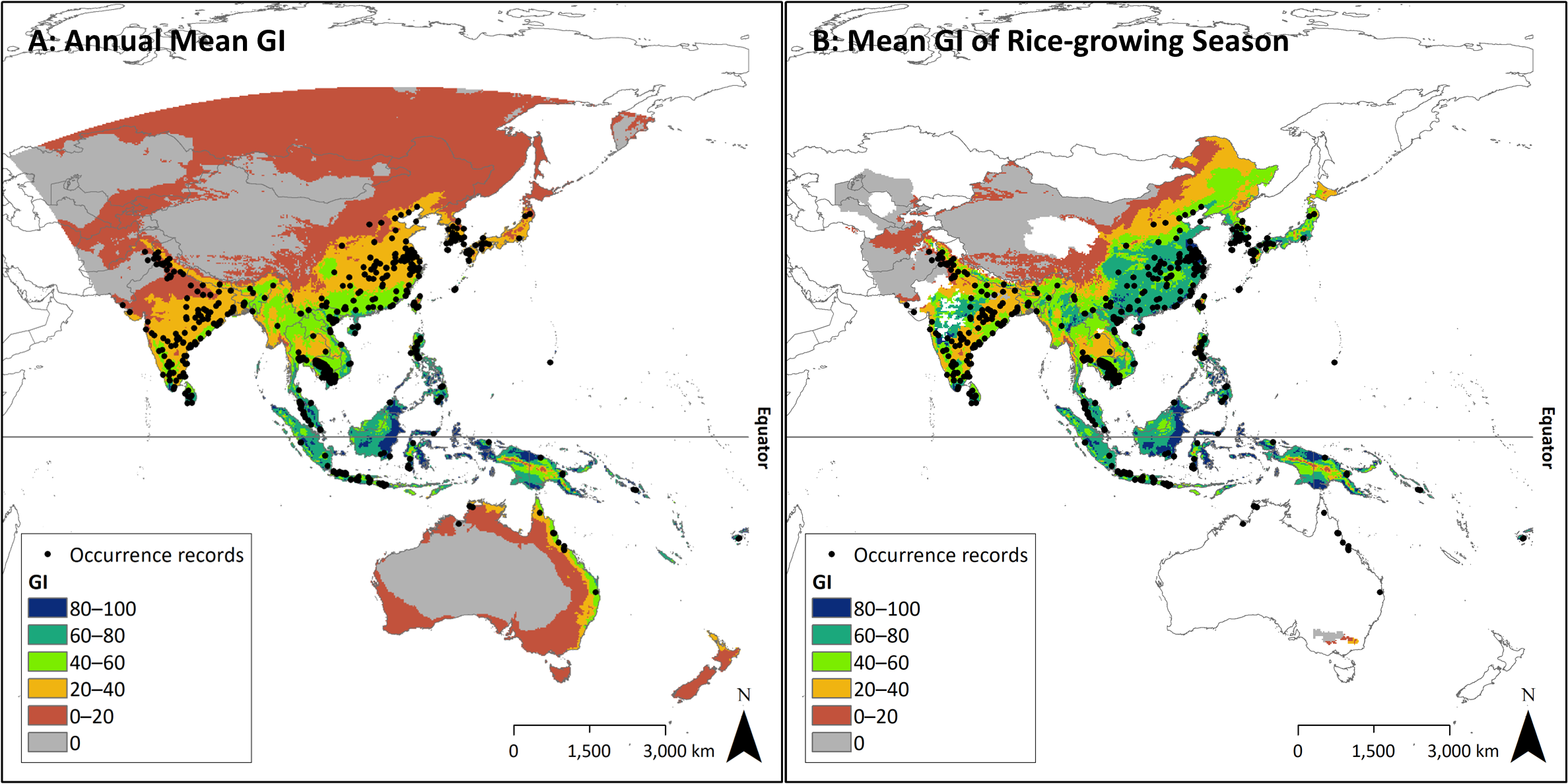


Supplementary 10. Growth Index of *Nilaparvata lugens* for annual (A) and rice-growing season (B) under the current climate. The rice-growing seasons are extracted from RiceAtlas dataset by summarizing ranges between peak planting day and peak harvesting day for each rice season.
